# Serum neurofilament light chain levels correlate with small fiber related parameters in patients with hereditary transthyretin amyloidosis with polyneuropathy (ATTRv-PN)

**DOI:** 10.1007/s10072-024-07562-0

**Published:** 2024-05-03

**Authors:** Eleonora Galosi, Rocco Costanzo, Francesca Forcina, Stefania Morino, Giovanni Antonini, Marco Salvetti, Antonio Lauletta, Marco Luigetti, Angela Romano, Guido Primiano, Valeria Guglielmino, Laura Fionda, Matteo Garibaldi, Nicoletta Esposito, Pietro Falco, Giuseppe di Pietro, Andrea Truini, Luca Leonardi

**Affiliations:** 1 Department of Human Neuroscience, Sapienza University of Rome, Rome, Italy; 2 Department of Neuroscience, Mental Health and Sensory Organs (NESMOS), Sapienza University of Rome, Rome, Italy; 3 Istituto Di Ricovero E Cura a Carattere Scientifico (IRCCS) Istituto Neurologico Mediterraneo Neuromed, Pozzilli, Italy; 4 Fondazione Policlinico Universitario A, Gemelli IRCCS, UOC Neurologia, Rome, Italy; 5 Dipartimento Di Neuroscienze, Università Cattolica del Sacro Cuore, Sede Di Roma, Rome, Italy; 6 UOC Neurologia, Sant’Andrea University Hospital, Rome, Italy

**Keywords:** Hereditary transthyretin amyloidosis (ATTRv), Small fiber neuropathy, Serum neurofilaments light chain, Skin biopsy, Quantitative sensory testing

## Abstract

**Background:**

Recent evidence suggests that both serum neurofilament light chain (sNfL) levels and small fiber related diagnostic variables may be valuable disease biomarkers of hereditary transthyretin amyloidosis with polyneuropathy (ATTRv-PN).

Our study aimed to explore the relations between sNfL and small fiber related skin biopsy and quantitative sensory testing (QST) parameters in a cohort of ATTRv-PN patients and pre-symptomatic carriers.

**Methods:**

We retrospectively analyzed data from 13 ATTRv patients and 21 pre-symptomatic carriers who underwent sNfL dosage, skin biopsy, and QST, and analyzed correlations between sNFL, intraepidermal nerve fiber density (IENFD), and cold (CDT) and warm detection thresholds (WDT).

**Results:**

Both sNfL and small fiber related parameters significantly differed between carriers and patients (sNfL: p < 0.0001; IENFD: p = 0.0008; CDT, WDT: < 0.0001). sNFL levels were normal in all carriers, altered in 85% of patients, negatively correlated with distal IENFD (r = -0.47, p = 0.005), and significantly correlated with CDT (r = -0.68; p < 0.0001) and WDT (r = 0.57; p < 0.0001).

**Conclusions:**

Our study showed that sNfL reliably discriminates symptomatic ATTRv-PN patients from pre-symptomatic carriers, and found significant relations between sNfL, skin biopsy, and QST small fiber related parameters, suggesting that sNfL might be a valuable biomarker of peripheral nerve involvement in ATTRv-PN and a supportive criterion for symptomatic disease transition.

**Supplementary Information:**

The online version contains supplementary material available at 10.1007/s10072-024-07562-0.

## Introduction

Hereditary transthyretin amyloidosis (ATTRv, v for ‘variant’) is an adult-onset, autosomal-dominant disease, caused by pathogenic variants in the TTR gene, encoding the transthyretin protein. Diffuse extracellular deposition of misfolded amyloidogenic mutant TTR causes multi-organ damage, with prevalent hearth and peripheral somatic and autonomic nerves involvement, leading to disability and death if left untreated [1]. Recently, the management of ATTRv has radically changed, thanks to the development of several therapeutic molecules able to delay or even prevent disease progression [2–5]. Since these therapies are greatly effective if early started in the disease course, efficient biomarkers of disease onset are increasingly needed [6].

In the last few years, small nerve fiber damage, as assessed by skin biopsy [6–14] and quantitative sensory testing (QST) [15–17], has been shown at early stages in ATTRv with polyneuropathy (ATTRv-PN), even preceding the onset of symptoms in pre-symptomatic carriers [14, 17]. At the same time, recent studies suggested that serum neurofilament light chain (sNfL), axonal structural compounds released during nerve degeneration, may help discriminate symptomatic patients from pre-symptomatic TTR mutation carriers [18, 19], thus representing a sensitive, non-invasive, and easily repeatable biomarker of disease onset [20–26].

Our study aimed to investigate the relations between sNfL, lately identified as promising disease biomarker, and small fiber related variables, which are well-known early impaired indicators of neural damage in the disease course. To do so, we retrospectively analyzed data from a cohort of ATTRv pre-symptomatic carriers and symptomatic patients who underwent both sNfL, skin biopsy, and QST assessment.

## Methods

### Subjects’ selection

**Fig. 1:**
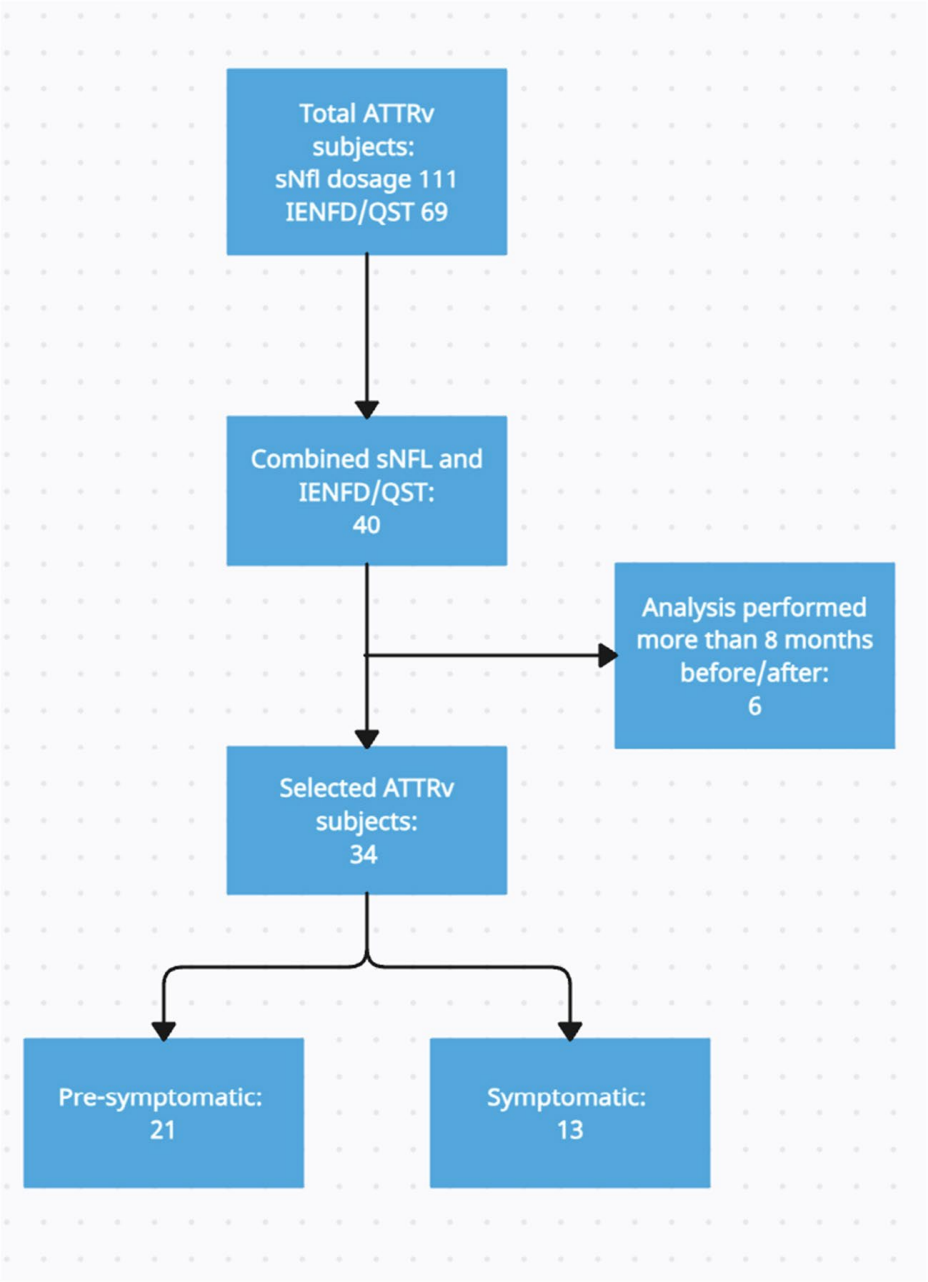
**Subjects’ selection flow-chart**. 140 ATTRv subjects were screened. From those 40 subjects who underwent both sNfL dosage, QST, and skin biopsy, we excluded 6 who had performed the procedures with a time-gap of more than 8 months before/after. We finally selected 34 ATTRv subjects, furtherly classified as pre-symptomatic carriers (n = 21) and symptomatic patients (n = 13)

We conducted a retrospective analysis of data from ATTRv patients and pre-symptomatic carriers participating to our two multicentric studies about small fiber dysfunction characterization through skin biopsy and QST [14, 17], who underwent sNfL dosage at different disease stages [18]. Subjects were recruited at our amyloidosis referral Centers (Sant’ Andrea and Agostino Gemelli University Hospitals) between January 2020 and November 2022, and were considered eligible if they carried a known amyloidogenic variant in TTR and were older than 18 years. Subjects with other known causes or risk factors for peripheral neuropathy, such as diabetes, alcohol abuse, vitamin B12 deficiency, paraproteinemia, or hypothyroidism, were excluded [18]. We selected subjects who underwent both nerve conduction study, skin biopsy/QST evaluation, and sNfL dosage in a timespan of ± 8 months (Fig. 1). We furtherly divided the study population into pre-symptomatic carriers and symptomatic patients. We considered as carriers those subjects with no sensory or vegetative symptoms, normal neurological examination with a Neuropathy Impairment Scale (NIS) of 0–1 [27], and normal sural sensory nerve action potential (SNAP) amplitude accordingly with our laboratories’ normal values. Conversely, subjects who reported at least two symptoms at the Small Fiber Neuropathy Symptoms Inventory Questionnaire (SFN-SIQ) [28] and presented a NIS ≥ 2 were considered as symptomatic patients [13], regardless the amplitude of sural SNAP. To avoid excluding patients with pure small fiber neuropathy from the study cohort, we did not incorporate a reduction in sural sensory nerve action potential (SNAP) as a criterion for symptomatic patients’ selection. Thus, symptomatic patients could exhibit either normal or impaired sural SNAP. Patients with normal sural SNAP fulfilled Besta criteria for small fiber neuropathy, based on clinical examination, QST, and skin biopsy findings [29]. Symptoms and signs associated with neurophysiological evidence of carpal tunnel syndrome (CTS) or following the median nerve's distribution were excluded from the calculation of SFN-SIQ and NIS scores. We exclusively included symptoms and signs indicative of polyneuropathy, characterized by a length-dependent or symmetrical distribution, in the determination of SFN-SIQ symptoms and NIS signs. Symptomatic patients were sub-classified using the polyneuropathy disability (PND) scoring system [30]. All subjects were routinely evaluated in our Centers by expert cardiologists to disclose any possible hearth involvement. Cardiac involvement was diagnosed in all individuals with increased interventricular septum thickness (> 13 mm) by echocardiography and/or the presence of amyloid deposits in the heart as assessed by Tc-99 m HMDP bone scintigraphy [31].

### Small nerve fiber assessment

Skin biopsy sampling, immunofluorescence staining protocol for protein gene product 9.5 (PGP 9.5) and collagen type IV, as well as intraepidermal nerve fiber density (IENFD) determination, were performed as previously reported [32]. Distal IENFD was considered as the main outcome skin biopsy variable using both raw values [33] and percentage reduction with respect to the lower cut-off of age-sex adjusted normative values [34].

QST was performed following the standardized protocol of the German Research Network on Neuropathic Pain [35]. The following QST small fiber related parameters were evaluated: cold and warm detection thresholds (CDT and WDT); cold and heat pain thresholds (CPT and HPT); mechanical pain threshold (MPT). A z-score was calculated for each QST variable (z-score = value of the patient—mean value of control subjects/standard deviation of control subjects) [36]. CDT and WDT were considered as main QST small fiber related outcome variables [37].

### sNfL measurement

Blood samples were collected into serum separator tubes, clotted for 30 min at room temperature and then centrifuged at 1000 g for 15 min. Aliquots were stored at − 80 °C until analysis. sNfL concentration was measured using the Simple Plex™ cartridge-based assay on the Ella™ platform (ProteinSimple, San Jose, CA, USA), according to the manufacturer's instructions. A cut-off of 37.10 pg/mL was considered for normalcy, as previously reported [26].

## Statistical analysis

A preliminary univariate analysis was performed to describe the main demographic and diagnostic test variables in pre-symptomatic carriers’ and patients’ group.

Since the normal distribution was rejected for all the considered continuous variables, as verified with the Kolmogorov–Smirnov test, quantitative variables were expressed as their median with interquartile range (IQR) and were compared between the two groups by the non-parametric Mann–Whitney U test. Correlations between the main outcome variables were verified through the Spearman’s correlation test. Simple linear regression models were used to assess linear relations between selected correlated variables.

Fisher’s exact test was used to compare the frequency of sNfL levels and small fiber related variables impairment between the two groups. For each variable, diagnostic specificity and sensitivity for the symptomatic disease state were calculated. Where applicable, simple logistic regression models were fitted to calculate the diagnostic accuracy of sNfL levels and small fiber related variables, as independent variables, to predict the presence of symptomatic disease state, as dependent variable.

No adjustment for multiple comparisons was adopted since all comparisons were pre-planned and our a priori intent was to test each variable independently. A p-value lower than 0.05 was considered significant. Analyses and graphics were performed with GraphPad Prism 10 software.

## Ethics approval

This protocol was approved by Sapienza University Ethical Committee (UNTTR-19–1, protocol number 275 SA_2019 19/12/2019) and Ethical Committee of Fondazione Policlinico Agostino Gemelli IRCSS (Prot. ID 4108). It was carried out according to the principles of the 1964 Declaration of Helsinki. Each enrolled subject provided a written informed consent, and all study data were obtained and elaborated in accordance with our institutional ethical committee regulations.

## Results

### Clinical and demographic variables

Subjects’ selection flow-chart is reported in Fig. 1. Briefly, 140 ATTRv subjects were screened. From those 40 subjects who underwent both sNfL dosage and small nerve fiber assessment, we excluded 6 who had performed the procedures with a time-gap of more than 8 months before/after. Finally, we selected 34 ATTRv subjects, furtherly classified as pre-symptomatic carriers (n = 21) and symptomatic patients (n = 13). Demographic and clinical variables of the two study cohorts are reported in Table 1. As expected, age differed significantly between patients and pre-symptomatic carriers. The p.Val50Met mutation was more represented in pre-symptomatic subjects, probably due to larger familiar clusters studied in our geographical area [30]. Most evaluated patients were classified as PND1, the milder stage of disease-related disability. Most patients showed a mixed phenotype, with both cardiac and neuropathic involvement, whereas 3 were cardiac prevalent and 2 neuropathic prevalent. All symptomatic patients had both increased interventricular septum thickness and heart amyloid deposit at the bone scintigraphy except from those three with neuropathic prevalent phenotype. All PND2 and PND3 patients were under treatment with Patisiran at the time of the evaluation. Out of 8 PND1 subjects, 5 were under disease-modifying treatments (2 Tafamidis, 2 Inotersen, 1 Patisiran).

**Table 1:** Demographic and diagnostic test variables in pre-symptomatic carriers and ATTRv patients with PN. Table 1 Continuous variables are expressed as median and interquartile range (IQR). Categorical variables are expressed as number of subjects (n) and percentages (%). Continuous variables were compared by Mann Whitney U test

	ATTRv patients (13)	Pre-symptomatic carriers (21)	P-value
Age (years)	73.5 (69.8–75.8)	45 (42–53.3)	< 0.0001
Onset (years)	67 (57.8–70)	-	-
Male/Female	8/5	12/9	-
p.Val50Met (n,%)	4 (31%)	15 (71%)	-
p.Phe84Leu (n,%)	5 (38%)	4 (19%)	-
Other mutations (n,%)	4 (31%)	2 (10%)	-
PND score	PND1 (8), PND2 (3), PND3 (2)	-	-
sNfl (pg/ml)	54.4 (37.1–76.0)	7.6 (4.3–16.7)	< 0.0001
Sural SNAP (μV)	5.5 (0.0–11.8)	16 (13.8–20.3)	< 0.0001
Distal IENFD (fibre/mm)	6.5 (3.3–8.2)	10.9 (8.5–14.7)	0.0008
% of axonal loss	-29 (-63.8 -10.3)	+ 14 (-20.0 -37.3)	0.0042
CDT (°C)	15.5 (0.0–25.9)	28.5 (16.8–30.7)	< 0.0001
z-score CDT	-2.417 (-3.692- -0.8613)	-0.7145 (-2.273- -0.0561)	0.0004
WDT (°C)	44.7 (37.4–50.0)	36.7 (33.8–43.3)	< 0.0001
z-score WDT	-1.607 (-2.073- -0.340)	-0.629 (-2.242–2.189)	0.0051
CPT (°C)	13.0 (0.0–38.0)	0.0 (0.0–16.9)	0.0190
HPT (°C)	50.0 (45.8–50.0)	45.0 (38.8–49.8)	< 0.0001
MPT (mN)	22.6 (5.6–181.0)	44.3 (7.8–1024.0)	0.0405

*PND* polyneuropathy disability score, *Sural SNAP* Sural sensory nerve action potential amplitude, *Distal IENFD* intraepidermal nerve fiber density from a distal site; % of axonal loss: percentage of IENFD reduction respect to the lower cut-off of age-sex adjusted normative values, *CDT* cold detection threshold, *WDT* warm detection threshold, *CPT* cold pain threshold, *HPT* heat pain threshold, *MPT* mechanical pain threshold

### Diagnostic test alterations distribution

Both sNfL levels and small fiber related parameters (distal IENFD, CDT, WDT, HPT, CPT, and MPT) significantly differed between pre-symptomatic and symptomatic subjects, as shown in Table 1. Sural SNAP was reduced or absent in 9 out of 13 (69%) of symptomatic patients. Symptomatic patients with normal sural SNAP fulfilled Besta criteria for small fiber neuropathy [29].

Considering a cut-off value of 37.10 pg/mL [26], sNfL levels were normal in all pre-symptomatic subjects and altered in 11/13 (85%) of symptomatic patients. Of the two symptomatic patients with normal sNfL levels, one had a cardiac prevalent phenotype (sNfL 28.4 pg/mL), the other one a mild mixed phenotype (sNfL 26.7 pg/mL). Fisher’s exact test showed that sNfL levels abnormalities could distinguish carriers from patients with a sensitivity of 84% and a specificity of 100% (p < 0.0001). A simple logistic regression model, with sNfL levels as independent variable and the presence of symptomatic disease state as dependent variable, showed that sNfL could significantly predict the disease state with an AUC of 0.99 (p < 0.0001).

IENFD was reduced in 9/22 (41%) of pre-symptomatic carriers and in 10/13 (77%) of symptomatic subjects. The main small fiber related QST outcome variables (WDT and/or CDT) were impaired in 4/21 (19%) of asymptomatic carriers and in 7/13 (54%) of symptomatic patients. Fisher’s exact test showed that the presence of IENFD reduction and of the main small fiber related QST variables abnormalities could not discriminate pre-symptomatic carriers from symptomatic patients.

### Correlations between sNfL and diagnostic variables

sNfL negatively correlated with distal IENFD (r = -0.47, p = 0.0050) and significantly correlated with the main small fiber related QST parameters impairment (CDT: r = -0.68, p < 0.0001; WDT: r = 0.57, p = 0.0005; HPT: r = 0.6, p = 0.0100; CPT: r = 0.44, p = 0.0002).

**Fig. 2:**
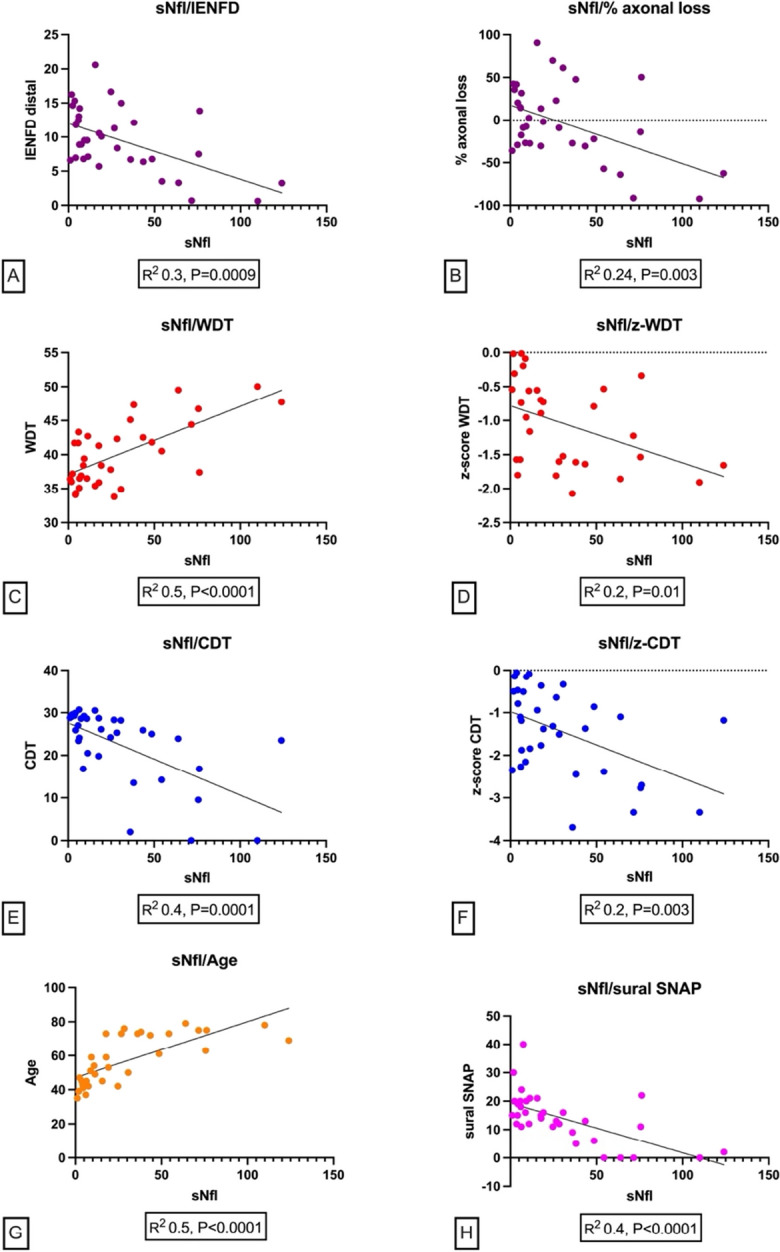
**Linear relations between sNfL levels and the main small fiber related variables**. Simple linear regression models showing a linear relation between sNfL and distal IENFD (**A**), WDT (**C**), CDT (**E**), and sural SNAP (**H**). Given the strict linear relation between sNfL and age (**G**), we also developed linear regression models using the percentage of axonal loss (**B**), i.e., the distal IENFD reduction respect to the lower cut-off of age-sex adjusted normative values, as well as the z-scores of WDT (**D**) and CDT (**F**), showing conserved linear relations between these variables. Legend: sNfL: serum neurofilament light chain levels; IENFD: intraepidermal nerve fiber density at the distal site; WDT: warm detection threshold; CDT: cold detection threshold; sural SNAP: sural sensory nerve action potential

Simple linear regression models showed a linear relation between sNfL and distal IENFD (r2 = 0.3, p = 0.0009), CDT (r2 = 0.4, p = 0.0001), WDT (r2 = 0.5, p < 0.0001), and sural SNAP (r2 = 0.4, p < 0.0001) (Fig. 2). Given the strict linear relation between sNfL and age (r2 = 0.5, p < 0.0001), we also developed linear regression models using the percentage of distal IENFD reduction respect to the lower cut-off of age-sex adjusted normative values (r2 = 0.2, p < 0.003), as well as the z-scores of CDT and WDT (r2 = 0.2, p < 0.003 and r2 = 0.2, p < 0.01 respectively), showing conserved linear relations between these variables (Fig. 2).

## Discussion

In our study we showed that sNfL reliably discriminates symptomatic ATTRv-PN patients from pre-symptomatic carriers, and found significant relations between sNfL, skin biopsy, and QST small fiber related parameters, suggesting that sNfL might be a valuable biomarker of peripheral nerve involvement in ATTRv-PN and a supportive criterion for symptomatic disease transition.

The therapeutic landscape of ATTRv deeply changed in the last decade, due to the advent of new effective disease modifying therapies (DMTs). Moreover, it has been demonstrated that the earlier a DMT is started, the better the functional outcomes are [38]. In this scenario, the development and validation of reliable biomarkers of disease onset are crucial to implement tempestive DMT initiation, avoiding inappropriate prescriptions.

Previous studies have shown significant changes in sNfL levels [20–24] and small nerve fiber function and morphometry [7–17], as assessed by skin biopsy and QST, since the earliest disease stages in several ATTRv-PN populations. However, no study has investigated the relation between sNfL and small fiber related parameters in ATTRv-PN to our knowledge.

NfL are structural compounds contributing to maintain mitochondrial and microtubule stability of neuronal soma and axons at both central and peripheral nervous system level, with increasing evidence demonstrating their rise in different nerve degeneration conditions, including polyneuropathies of various etiologies [39–42].

Small fiber damage has traditionally been considered a hallmark of ATTRv-PN and has been early detected even in pre-symptomatic mutations carriers with skin biopsy and QST thermal thresholds assessment, the most widely agreed tools to assess small fiber morphometry and function [37, 43, 44]. In our cohort, small fiber related parameters abnormalities were unable to reliably distinguish pre-symptomatic from symptomatic subjects, thus showing a low specificity for the disease state condition; in fact, consistently with previous data [17, 45], distal IENFD and QST thermal thresholds resulted to be frequently early impaired also in pre-symptomatic carriers, with 41% showing distal IENFD reduction and 19% QST abnormalities of CDT and/or WDT.

Conversely, in our study sNfL levels abnormalities could predict the presence of symptomatic disease with very high sensitivity (85%) and specificity (100%), with all pre-symptomatic carriers showing normal sNfL levels (< 37.10 pg/mL). Since sNfL increase in peripheral neuropathies has been linked to peripheral nerve damage acuteness and progression [41, 42], we could speculate that small fiber damage, even if early detectable at skin biopsy and QST, occurs at such a mild rate and degree in pre-symptomatic subjects that it doesn’t induce an appreciable rise of sNfL, until a hypothetical threshold is reached when both neuropathic symptoms and sNfL changes begin. In this respect, sNfL rise could represent a supportive criterion to diagnose symptomatic transition in ATTRv carriers, in addition to the traditional small fiber related diagnostic parameters like distal IENFD and QST thermal thresholds, whose specificity is lowered by early abnormalities in asymptomatic carriers.

Noticeably, most symptomatic patients of our study cohort were staged as PND1, representing the earlier disease phase, exclusively characterized by sensory and autonomic neuropathic symptoms [46]. This could explain why median sNfL levels in our patients appear to be lower than previously reported [21, 22] (Table 1).

As previously shown [13], a consistent proportion of these PND1 symptomatic subjects has normal NCS, making the instrumental demonstration of polyneuropathy onset challenging. In our study, the combination of sNfL (using a cut-off of 37.10 pg/mL) with distal IENFD and QST thermal thresholds (CDT and/or WDT), permitted to document neuropathic damage abnormalities in all symptomatic patients, in all cases in combination (e.g. elevated sNfL and reduced distal IENFD), thus remarking the potential usefulness of sNfL as a supportive criterion to identify symptomatic transition.

In our study sNfl levels significantly correlate with distal IENFD and the main small fiber related QST parameters, i.e. CDT and WDT. These correlations, corroborated by linear regression models, persisted even when the effect of the age was marginalized using age-adjusted normal values for distal-IENFD and z-score for QST parameters (Fig. 2). sNfl levels also correlated with sural SNAP; however, this correlation might be mostly driven by affected individuals, since in our study all the carriers had normal sural SNAP, as per selection criteria, whereas most symptomatic patients had abnormal sural SNAP.

Increasing knowledge suggests that sNfL levels are elevated in polyneuropathies at early stages [26]. A recent report showed sNfL levels increase even in patients with pure small fiber damage without large fiber neuropathy, leading the authors to propose sNfL as a surrogate biomarker for small fiber involvement detection in ATTRv with PN [47]. Conversely, other studies didn’t find any difference in sNfL levels between patients with small fiber neuropathy of various etiologies and healthy subjects, thus questioning the role of sNfL as a small fiber damage biomarker [48]. A plausible explanation for the inconsistency between findings in ATTRv-PN and small fiber neuropathies of other etiologies, may lie in the fact that small fiber neuropathy in ATTRv represents the detectable aspect of a broader spectrum of progressive damage, probably originating at the dorsal root ganglia level [14, 49, 50]. This hypothesis could elucidate why sNfL levels are elevated in ATTRv, even when patients exhibit symptoms solely attributable to small fiber neuropathy.

Considering that skin biopsy, the gold standard for small fiber assessment [43, 51], is not universally available, is time-demanding and minimally invasive, the elaboration of alternative, easier to perform biomarkers of small fiber damage is warranted and represents a compelling clinical challenge. Based on our results, we cannot assume that sNfL is a selective biomarker of small fiber degeneration in ATTRv-PN, since we found significant correlations between sNfL levels and large fiber mediated variables like sural SNAP amplitude. However, the correlations we found between sNfL and small fiber damage related parameters, which are early impaired in the disease course, confirm the potential usefulness of sNfL as a biomarker of nerve degeneration in ATTRv-PN.

## Limitations

Even though small fiber damage assessment was performed according to widely agreed recommendations and only patients who underwent sNfL determination close to small fiber assessment were selected, the retrospective design may be considered as a limitation of our study. Further prospective, longitudinal, and follow up-validation studies are needed to confirm the potential yield of sNfL as a disease biomarker in ATTRv-PN. Another important limitation of our study is the relatively small sample size, since ATTRv-PN is a rare disease. However, we believe that our sample reliably reflects late-onset ATTRv-PN carriers’ and patients’ characteristics in our local population, due to age uniformity and strict selection criteria.

Admittedly, the clinical characteristics of our cohort, predominantly comprising patients with a mixed ATTRv phenotype, precluded us from drawing definitive conclusions regarding patients with prevalent cardiac involvement. Notably, we acknowledge the possibility that sNfL might not effectively identify the transition to symptomatic status in patients with dominant cardiac involvement, as evidenced by the normal sNfL levels observed in the three patients with a predominant cardiac phenotype in our study.

Moreover, the criteria utilized for distinguishing pre-symptomatic carriers from symptomatic patients may impact our findings regarding the efficacy of sNfL to distinguish between these groups. Specifically, we categorized patients as symptomatic if they reported at least two symptoms on the SFN-SIQ and had a NIS of ≥ 2, regardless of sural SNAP amplitude [13]. This approach could be viewed as more inclusive compared to previously published criteria [52]. However, we opted to include patients with normal sural SNAP in the symptomatic group only if they met the Besta criteria for definite small fiber neuropathy based on clinical, quantitative sensory testing (QST), and skin biopsy abnormalities [29].

## Conclusions

Our study showed that sNfL dosage reliably discriminates symptomatic ATTRv-PN patients from pre-symptomatic carriers, and found significant relations between sNfL levels, skin biopsy, and QST small fiber related parameters, thus suggesting that sNfL might be a valuable biomarker of peripheral nerve involvement in ATTRv-PN and a supportive criterion to identify symptomatic disease transition.

## Supplementary Information

Below is the link to the electronic supplementary material.

Supplementary file1 (DOCX 236 KB)

## Data Availability

Data related to this study are available upon reasonable request.

## Abbreviations

ATTRv-PN: Hereditary transthyretin amyloidosis with polyneuropathy
CDT: Cold detection threshold
CPT: Cold pain threshold
DMTs: Disease modifying therapies
HPT: Heat pain threshold
IENFD: Intraepidermal nerve fiber density
MPT: Mechanical pain threshold
NIS: Neuropathy Impairment Scale
PND: Polyneuropathy disability scoring system
QST: Quantitative sensory testing
SFN-SIQ: Small Fiber Neuropathy Symptoms Inventory Questionnaire
SNAP: Sensory nerve action potential
SNfL: Serum neurofilament light chain
TTR: Transthyretin
WDT: Warm detection threshold
