# Supplementary material for: Serum neurofilament light chain levels correlate with small fiber related parameters in patients with hereditary transthyretin amyloidosis with polyneuropathy (ATTRv-PN)
Source: Neurol Sci. 2024 May 3;45(10):5023–32. doi: 10.1007/s10072-024-07562-0 (PMC11422273; doi:10.1007/s10072-024-07562-0)
Supplement: Supplementary file 1 — Supplementary file1 (DOCX 236 KB) [file 10072_2024_7562_MOESM1_ESM.docx]

**Supplementary Table 1. Correlation matrix showing Spearman’s r coefficients for the main diagnostic test variables.**


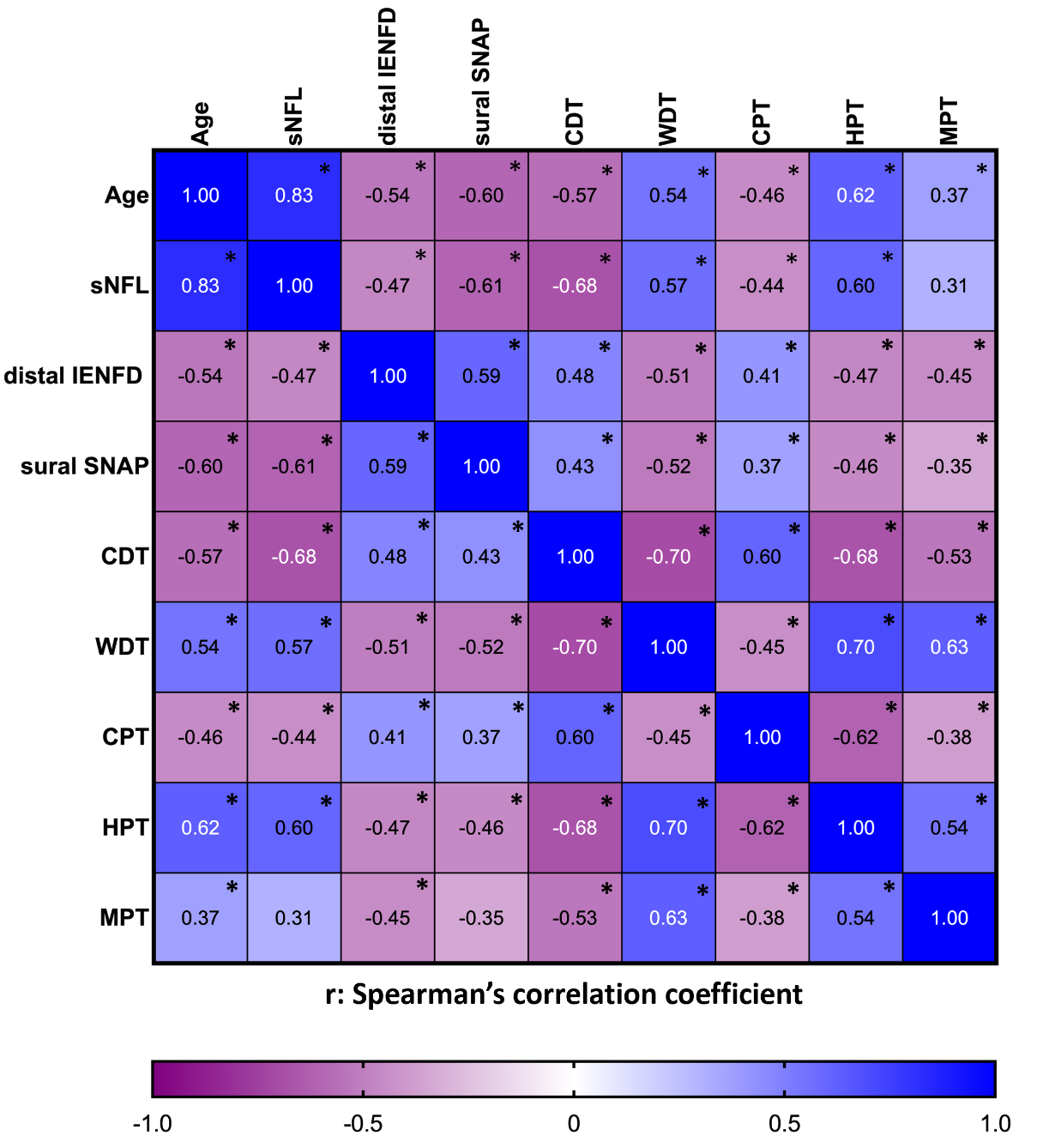


Supplementary Table 1. * indicate correlations with p<0.05. sNfL: serum neurofilament light chain levels; distal IENFD: intraepidermal nerve fibre density at the distal site; sural SNAP: sural sensory nerve action potential; CDT: cold detection threshold; WDT: warm detection threshold; CPT: cold pain threshold; HPT: heat pain threshold; MPT: mechanical pain threshold.
